# Systemic and brain delivery of antidiabetic peptides through nasal administration using cell-penetrating peptides

**DOI:** 10.3389/fphar.2022.1068495

**Published:** 2022-11-14

**Authors:** Jeehye Maeng, Kyunglim Lee

**Affiliations:** Graduate School of Pharmaceutical Sciences, College of Pharmacy, Ewha Womans University, Seoul, South Korea

**Keywords:** protein transduction domain, cell-penetrating peptide, PTD, CPP, TCTP-PTD, nose-to-brain delivery, nasal delivery, antidiabetic peptides

## Abstract

The intranasal route has emerged as a promising strategy that can direct delivery of drugs into the systemic circulation because the high-vascularized nasal cavity, among other advantages, avoids the hepatic first-pass metabolism. The nose-to-brain pathway provides a non-invasive alternative to other routes for the delivery of macromolecular therapeutics. A great variety of methodologies has been developed to enhance the efficiency of transepithelial translocation of macromolecules. Among these, the use of cell-penetrating peptides (CPPs), short protein transduction domains (PTDs) that facilitate the intracellular transport of various bioactive molecules, has become an area of extensive research in the intranasal delivery of peptides and proteins either to systemic or to brain compartments. Some CPPs have been applied for the delivery of peptide antidiabetics, including insulin and exendin-4, for treating diabetes and Alzheimer’s disease. This review highlights the current status of CPP-driven intranasal delivery of peptide drugs and its potential applicability as a universal vehicle in the nasal drug delivery.

## 1 Introduction

Successful delivery of drugs at a sufficient concentration to the target cells is critical in achieving the desirable therapeutic effects (Xie et al., 2020). The cell membrane that functions as a physical barrier, limits the intracellular delivery of therapeutics, especially peptide, protein, nucleic acid, and nanocarrier drugs to their molecular targets. Therefore, there is a need for vehicles that can transport drugs across cell membranes.

This need was recently addressed with the identification of a group of specific peptides that can transport small molecules as well as hydrophilic macromolecules into the cell interior (Szabó et al., 2022). The growing family of such peptides has been called cell-penetrating peptides (CPPs) or protein transduction domains (PTDs). These peptides have proven to be versatile vectors that have emerged as promising vehicles that have overcome the low permeability of macromolecular therapeutics through the epithelial membrane for transmucosal delivery by noninvasive and convenient methodology. CPPs have been proven to successfully deliver covalently or non-covalently bound cargos into the live cells and through the epithelial tissues, including the nasal cavity.

This review focuses on the application of nasal delivery of drugs using CPP-based systems based on our current understanding of how CPP can be applied *in vivo* delivery of peptide drugs through the nasal epithelium and of the potential advantages of the nasal administration route in systemic and brain drug delivery. The present review also highlights the value of the CPP-driven drug delivery in the perspective of novel transduction platforms, more specifically for the nasal antidiabetic drug delivery.

## 2 Cell-penetrating peptides as novel carrier molecules

CPPs, usually 5-30 amino acids-long peptide moieties (Kurrikoff et al., 2021), are not only able to pass through the plasma barrier but also capable of facilitating the cellular transport of drugs and other conjugated or coadministered cargos (Liu et al., 2022). Such CPP/cargo complexes have been studied for their potential applications in the diagnosis and treatment of human diseases at basic and preclinical levels, predominantly directed at the therapy of inflammation, cancer, diabetes, central nervous system (CNS) disorders, and ocular and otologic disorders (Xie et al., 2020).

Most CPPs are identified from unexpected finding that specific full-length proteins can cross the cell membrane. The small translocating motif within the protein that is responsible for the translocation is delineated by truncation or mutational analysis of the parent protein (Joliot and Prochiantz, 2004). The transduction ability of CPPs suggests that the full-length protein can move in and out of cells as a part of some specific physiological processes (Joliot and Prochiantz, 2004). The most extensively studied CPP was first identified in 1988 from the observations of the cellular translocating ability of human immunodeficiency virus (HIV) transactivator of transcription (TAT) protein that is responsible for viral replication (Frankel and Pabo, 1988; Green and Loewenstein, 1988). TAT protein enters the nucleus after being taken up by the cells, and in turn induces the expression of target genes serving as a viral growth factor (Frankel and Pabo, 1988; Green and Loewenstein, 1988). Later studies identified several basic peptide domains, such as residues 48-60 (Vivès et al., 1997) or 48-56 (Chauhan et al., 2007), which are mapped to the cell-penetrating moieties of the full-length TAT protein. Its cargo-delivering activity was demonstrated both *in vitro* and *in vivo* (Nagahara et al., 1998; Schwarze et al., 1999). Another CPP originating from the homeodomain of antennapedia, pAntp, was reported to internalize the nerve cells and to induce morphological differentiation (Joliot et al., 1991). A subsequent study by Derossi *et al.* showed that a 16 amino acids-long peptide fragment from the third helix of the antennapedia homeodomain called penetratin, is the membrane translocating moiety (Derossi et al., 1994).

Since then, various CPPs with different physicochemical properties in terms of length, charge, hydrophobicity, and solubility have been identified and characterized from both natural and artificial origins (Xie et al., 2020). Such CPPs are generally classified by their origins (protein-derived, chimeric, and synthetic), physicochemical properties (cationic, amphipathic, and hydrophobic), and conformations (linear and cyclic) (Xie et al., 2020). The majority of them are linear, synthetic, protein-derived, and amphipathic CPPs (Xie et al., 2020). Cationic (TAT-PTD, Penetratin, polyarginine, etc.) and amphipathic CPPs (MPG, Pep-1, pVEC, MAP, etc.) constituted about 85% of the total CPPs, whereas only 15% belonged to hydrophobic CPPs (TCTP-PTD, Pep-7, FGF, etc.) (Mickan et al., 2014; Kang et al., 2019; Zhang et al., 2021).

The internalizing mechanism of CPPs is largely dependent on the properties of CPPs, cell types (membrane compositions and cellular machineries for uptake), transported cargos (concentration, structure, and linking methods), and the experimental conditions (pH and temperature) (Xie et al., 2020). The primary translocation mechanisms utilized by CPPs are direct translocations or endocytosis, according to the energy dependency of the internalization process (Zorko and Langel, 2022). Most CPPs are translocated *via* the plasma membrane through direct translocation or through the interaction of CPPs with the cell surface components that drive endocytic uptake. Direct translocation is an energy-independent and non-endocytic pathway, which is motivated by the initial interactions of CPPs with the constituents of lipid bilayers. During this process, hydrogen bonding or electrostatic interaction induces the destabilization of the membrane or formation of pores for the translocation (Xie et al., 2020). On the other hand, energy-dependent endocytosis is predominantly used for the cellular uptake of large CPPs or large CPPs/cargo complexes, which involves caveolin-mediated endocytosis, clathrin-mediated endocytosis, clathrin/caveolin-independent endocytosis, and macropinocytosis (Szabó et al., 2022). After endocytic internalization, the fate of CPP/cargo complexes primarily relies on their ability for endosomal escape before their transport into the endosome, a site for enzymatic degradation (Dinca et al., 2016).

Use of CPPs enables the noninvasive delivery of macromolecular drugs, such as nucleic acids, proteins, imaging agents, viruses, and CPP/cargo complexes through various mechanisms of transduction pathways without perturbing the membrane integrity (Borrelli et al., 2018). Currently, several CPP-based therapeutics have been under clinical trials for their potential applications in the therapy and diagnostics of human diseases, such as solid tumor, glioma, CNS tumor, and muscular dystrophy (Xie et al., 2020; Kurrikoff et al., 2021).

Moreover, mounting evidence has demonstrated the feasibility of several types of CPPs, including TAT-PTD, TCTP-PTD, polyarginine, and penetratin and analogs thereof (Table 1), in the transepithelial delivery through nasal and intestinal epithelium (Kamei et al., 2008; Khafagy et al., 2010a, 2012; Kamei et al., 2013a; Bae and Lee, 2013; Bae et al., 2018a) and of their potential for use as advanced drug delivery vehicles (Zhu et al., 2014; Shan et al., 2015; Zhang et al., 2015; Kristensen and Nielsen, 2016). Although the exact mechanism by which CPPs translocate the epithelium is not fully elucidated, these motifs are regarded as promising vectors for transepithelial transport and the basis for developing more advanced drug delivery strategies (Kristensen and Nielsen, 2016).

**TABLE 1:** Examples of CPPs described in the present article. The theoretical isoelectric point and molecular weight were obtained using the ExPasy website (https://web.expasy.org/compute_pi/).

PTD	Sequence	pI	M.W	Length	Origin	Reference
Hydrophobic						
TCTP-PTD	MIIYRDLISH	6.50	1,261	10	Human TCTP protein	Bae and Lee, (2013)
TCTP-PTD 13	MIIFRALISHKK	11.17	1,457	12	TCTP-PTD analog	Bae and Lee, (2013)
TCTP-PTD 13M2	MIIFRLLASHKK	11.17	1,457	12	TCTP-PTD analog	Bae et al. (2018a); Bae et al. (2018b)
TCTP-PTD 13M3	MIIFRLLAYHKK	10.29	1,533	12	TCTP-PTD analog	Bae et al. (2018a)
Amphipathic						
Penetratin	RQIKIWFQNRRMKWKK	12.31	2,247	16	*Drosophila* homeodomain	Khafagy et al. (2009a); Khafagy et al. (2009b)
Shuffle (R,K fix) 2	RWFKIQMQIRRWKNKK	12.31	2,247	16	Penetratin analog	Khafagy et al. (2010b)
PenetraMax	KWFKIQMQIRRWKNKR	12.31	2,247	16	Penetratin analog	Khafagy et al. (2013)
Cationic						
TAT-PTD	CGGGYGRKKRRQRRR	12.01	1,834	15	HIV transcription activator	Liang and Yang, (2005)
	RKKRRQRRR	12.70	1,340	9	HIV transcription activator	Chauhan et al. (2007)
R8 (Octaaginine)	RRRRRRRRR	12.90	1,424	8	Artificial peptide	Khafagy et al. (2009a)

## 3 Feasibility of CPP-mediated nasal drug delivery

### 3.1 Characteristics of nasal administration route in the drug delivery

Mucociliary clearance refers to the clearance of the mucus and substances that are adsorbed in the nasal cavity or dissolved in the mucus, pushing it into the gastrointestinal tract through nasopharynx (Ozsoy et al., 2009). Such physiological clearance mediated by the hair-like cilia on the surface of epithelial cells with their fluctuating movement is responsible for the clearing of nasally administered drugs from the nose with a half-life of 21 min (Ozsoy et al., 2009). Compared to other routes of drug administration, nasal mucosa has a relatively porous and thin basement membrane (Bajracharya et al., 2019). Nasal mucosa offers a large surface area for the molecular absorption because of its highly vascularized subepithelial tissues and can direct the delivery of nasally administered molecules into the systemic circulation (Khafagy et al., 2009a). Moreover, intranasal pathway can avoid the first-pass metabolism by the liver, thereby enhancing the bioavailability of drugs (Erdő et al., 2018). Rapid nasal absorption of drugs facilitates quick onset of pharmacological effects (Montegiove et al., 2022). The nasal route has the advantage of self-medication by patients due to the easy accessibility of the nasal cavity (Khafagy et al., 2009a). Most intranasal drugs used to treat local inflammation of the nasal mucosa are small molecules such as steroids. Some small peptides, such as oxytocin and vasopressin, as well as some vaccines are administered *via* nasal routes for systemic effects (Ozsoy et al., 2009). Currently, nasal route has attracted attention as an alternative to the invasive administration of insulin injection (Pontiroli et al., 1989; Ozsoy et al., 2009; Chen et al., 2018).

The choice of nasal route for drug administration is mainly determined by considering the physicochemical properties of drugs, including hydrophobicity and molecular size (Behl et al., 1998). The bioavailability of nasally administered hydrophilic peptides and proteins, particularly for those with a size of >1,000 Da, is very low because of their weak permeability and susceptibility to proteolysis in the nasal mucosa (McMartin et al., 1987). The inverse relationship between the molecular weights of proteins or peptides and efficiency of nasal absorption is well established (Pires et al., 2009). Pseudo-first-pass effects that degrade drugs in the nasal mucosa by the action of diverse enzymes also hinder the nasal absorption (Sarkar, 1992). Taken together, the major hindrances for nasal delivery include low absorption of large molecules, possible enzymatic degradation in the nasal mucosa, and the clearance by rapid action of mucociliary transport (Khafagy et al., 2009a; Ozsoy et al., 2009), all of which contribute to the low bioavailability of drugs. In addition, the limit of volumes (25–250 μl) for nasal administration, influence of nasal pathologic conditions such as rhinitis and common cold on the absorption, and potential for nasal irritation by drug administration in the nasal cavity, are significant factors governing in the choice of the nasal route for drug application (Ozsoy et al., 2009; Kashyap and Shukla, 2019).

To overcome the above-mentioned hurdles of intranasal drug delivery, peptides and proteins can be modified to increase their membrane permeability and stability, by supplementing the drug with enzyme inhibitors that perturb the pseudo-first-pass effects (Pires et al., 2009). Absorption enhancers may also promote the uptake of drugs through the nasal mucosa and advanced formulations using liposome, lipid emulsions, and nano- and micro-particles can be used to optimize intranasal drug delivery (Ozsoy et al., 2009). Nano- and micro-particle systems, matrix systems where the molecules are distributed in the polymeric structures, facilitate sufficient retention of drugs in the nasal cavity and the protection against enzymatic activity. It also mediates tight contact between drug and nasal mucosa and even promotes the opening of tight junctions (Ozsoy et al., 2009). To attain sufficient bioavailability for nasally administered proteins and peptides, absorption enhancers, including cyclodextrin, surfactants, and mucoadhesive polymers have been applied in many formulation studies (Khafagy et al., 2009a).

### 3.2 Pathways of transepithelial transport

**FIGURE 1:**
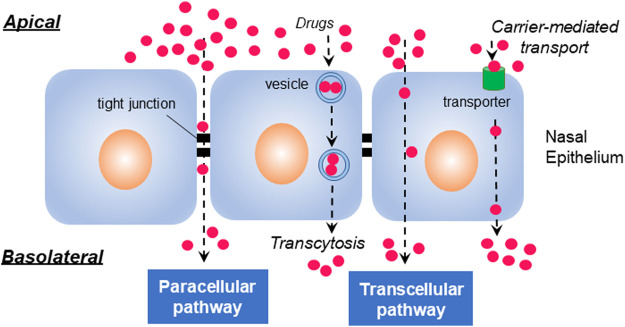
Transepithelial pathways. Molecules generally penetrate the epithelial cells through paracellular, transcellular, and transcytotic pathways. The paracellular pathway is used by drugs to passage through intermolecular spaces or tight junctions of epithelium, whereas transcellular translocation is mediated by passive diffusion, carrier-mediated transport, or endocytosis. Transcytosis occurs by vesicular transduction of cargos.

To reach the bloodstream on the capillaries, drugs have to pass through the mucus barrier. Subsequently, drugs are known to follow the normal pathways for transepithelial transport (Figure 1). Transepithelial transport can occur by energy-independent direct uptake or paracellular diffusion, and by energy-dependent endocytic pathways and transcytosis, receptor-aided transport, or carrier-medicated translocation (Kristensen and Nielsen, 2016).

Transcellular transport mediated by either passive diffusion or active transport is a cellular process primarily used for the transport of lipophilic molecules (Ozsoy et al., 2009). Paracellular transport, a slow and passive aqueous pathway (Fortuna et al., 2014) that is accomplished by the regulation of tight junctions and passage through the intercellular spaces between adjacent cells, is important for the transepithelial absorption of drugs (Lemmer and Hamman, 2013). The localized high concentration of CPPs may influence the integrity of tight junctions, subsequently affecting the transduction of cargos through the paracellular cleft (Kristensen and Nielsen, 2016). It was found that some CPPs, such as transportan, MAP (model amphipathic peptide), and L-R5, reversibly modulate tight junctions in the transepithelial transport (Lindgren et al., 2004; Bocsik et al., 2019; Ragupathy et al., 2021). The transcytotic pathway is used for the vesicular transport of macromolecules from the apical to basolateral or the reverse, depending on the cargos and cellular processes in epithelial cells (Tuma and Hubbard, 2003). After the endocytic uptake of cargo with CPPs, transepithelial transport can be accomplished *via* transcytosis, avoiding lysosomal degradation (Kristensen and Nielsen, 2016).

Among the passage routes of drugs *via* nasal mucosa, large molecules like peptides, proteins, and nucleic acids are often reported to cross the nasal epithelium following the transcellular pathway or endocytosis (Illum, 2003; Ozsoy et al., 2009). In contrast, small molecules internalize the epithelium *via* the paracellular pathway through intercellular tight junctions (Ozsoy et al., 2009). Also found is that transepithelial transport of macromolecules such as protein and peptide therapeutics is highly limited (Bruno et al., 2013).

### 3.3 Challenges of CPP-mediated nasal drug delivery

Although CPP-driven nasal delivery is regarded as a promising tool in facilitating non-injectable delivery of drugs, there are some challenges. First, CPPs in the nasal cavity are susceptible to enzymatic degradation in the mucus and the surface of the epithelium because of the inherent physicochemical characteristics of peptides (Kaur et al., 2016). The chemical modification of CPPs, peptide cyclization, and its PEGylation can improve the metabolic stability of CPPs (Szabó et al., 2022). In addition, as for CPP-mediated nose-to-brain delivery, the systemic exposure of nasally administered drugs might also occur because nasal coadministration of peptides with CPPs is shown to transport the peptide drugs to both systemic and brain compartments. Pharmacokinetic studies can further develop the specified methodologies for targeted delivery of drugs from the nasal cavity to specific compartments.

Recently, innovative nasal drug delivery systems have been developed to maximize its bioavailability by enhancing the solubility, permeability, and retention time at the nasal mucosa. For example, specific formulations have been investigated for nasal drug delivery, such as soft nano-vesicular systems, microsphere/inert carrier dry powder platform for brain-targeted delivery, and binary composite solid microparticles as nasal permeation enhancers (Hafner, 2022). Unlike these strategies, CPPs *per se* are able to internalize live cells and are peptide enhancers that mediate the transepithelial transport of large molecules without affecting cellular integrity. In addition, CPPs can be applied in the combination of the above-mentioned drug delivery platforms, enhancing the feasibility of CPP-aided nasal drug delivery.

## 4 Systemic and brain delivery through nasal administration

**FIGURE 2:**
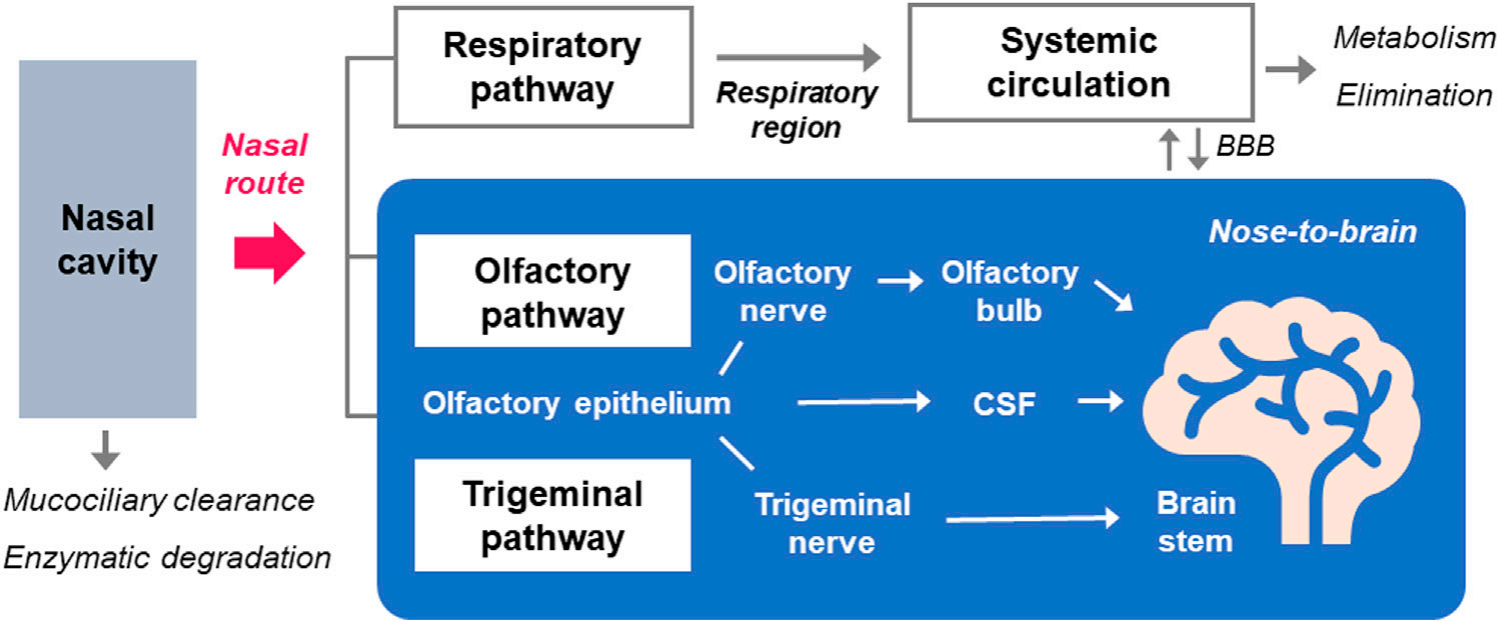
The routes for the systemic and nose-to-brain delivery of molecules by nasal administration. Following the nasal administration of drugs, some are subjected to mucociliary clearance and enzymatic actions in the nasal mucosa before their entry into systemic and CNS compartments. Drugs in the respiratory region around the inferior turbinate in the nasal cavity are absorbed into the blood circulation through the respiratory pathway. Nose-to-brain pathway is largely divided into olfactory and trigeminal pathways, which involve axonal transport of olfactory and trigeminal nerves, respectively. Transepithelial permeation of drugs across the olfactory epithelium also contributes to its delivery to the brain after nasal administration, through the CSF distribution.

The nasal cavity is functionally divided into three distinctive areas, namely, respiratory, olfactory, and vestibule regions (Ozsoy et al., 2009; Selvaraj et al., 2018). The surface area of the nasal cavity is about 160 cm^2^ in humans (Gizurarson, 2012). The nasal vestibule, the most anterior part of the nasal cavity, is not involved in the drug absorption, whereas the respiratory region is the main part for drug absorption due to its large surface area (Selvaraj et al., 2018). The olfactory region is constituted with olfactory receptor cells that transduce the neuronal signal from the epithelium to olfactory bulb, and other cells, including sustentacular and basal cells (Selvaraj et al., 2018). The nasal drug administration can deliver the drugs either to the local nasal cavity or to systemic circulation through the respiratory region of nasal cavity (Ozsoy et al., 2009). In addition, some drugs follow the nose-to-brain pathway involving olfactory and trigeminal routes through the superior olfactory region and the lateral respiratory region, respectively (Figure 2).

### 4.1 Systemic transport after nasal administration

Nasal administration of drugs can direct the rapid systemic absorption of drugs by circumventing the hepatic first-pass metabolism and gastric degradation, allowing fast onset of pharmacological action (Tai et al., 2022). In general, larger particles more than 10 μm accumulate in the respiratory area, whereas particles smaller than 5 μm can reach the lungs (van Rijt et al., 2014). Most administered drugs by the nasal route can be absorbed in the respiratory area of the nasal cavity enriched with vasculature throughout the extensive surface area (Ozsoy et al., 2009). The respiratory region around the inferior turbinate is the major site for systemic entry of nasal drugs due to its greatest surface area in the nasal cavity (Fortuna et al., 2014). Blood supply to the nasal mucosa and its flow rate are critical determinants for the systemic absorption of drugs to the blood circulation *via* diffusion (Fortuna et al., 2014).

### 4.2 Nose-to-brain transport after nasal administration

Blood-brain barrier (BBB) is constructed through the networking of brain capillary endothelial cells that are held together by tight junctions, which segregates the blood perfusion into the CNS (Hallschmid, 2021). It serves as a physiological or metabolic barrier that imposes highly selective permeation of molecules into the CNS (Daneman and Prat, 2015; Ross et al., 2018), although it perturbs the delivery of neurotherapeutics (Pardridge, 2003). The factors that restrict the influx of drugs include membrane barrier, inter-endothelial cell tight junctions, efflux transporters, including p-glycoprotein (Cioni et al., 2012) and degrading enzymes of BBB (Ross et al., 2018).

Currently, intranasal delivery is suggested as a promising alternative to invasive strategies because it can directly transport the molecules to the brain bypassing the BBB (Crowe et al., 2018; Erdő et al., 2018; Kashyap and Shukla, 2019). The intranasal route has been found to be effective in delivering the therapeutic molecules by avoiding liver metabolism, renal filtration, elimination in the gastrointestinal tract, and degradation in the serum (Mistry et al., 2009; Malhotra et al., 2013). Anatomically, nasal mucosa is directly connected to the cerebrospinal fluid (CSF) and parenchymal regions of the brain (Dhuria et al., 2010; Lochhead and Thorne, 2012; Crowe and Hsu, 2022). Nose-to-brain pathway was found to be efficient in the delivery of bioactive peptides and growth factors, such as exendin-4, leptin, orexin, vasoactive intestinal peptide (VIP), galanin-like peptide (GALP), interferon-β, insulin-like growth factor (IGF), nerve growth factor (NGF), brain-derived neurotrophic factor (BDNF), and basic fibroblast growth factor (bFGF) (Kamei et al., 2017). Furthermore, nose-to-brain transport of small molecular drugs, proteins, siRNA, and stem cells *in vivo*, and of peptide drugs, including insulin, vasopressin, melanocortin, angiotensin II, and oxytocin in humans was successfully accomplished (Lalatsa et al., 2014; Kanazawa, 2015).

The transport of molecules after the absorption in the nasal cavity is known to follow multiple pathways (Dhuria et al., 2010). The olfactory region, more specifically the olfactory bulb, is the only compartment throughout the human body where peripheral regions keep in direct contract with the CNS (Selvaraj et al., 2018). This olfactory epithelium directs the brain delivery of drugs *via* olfactory and trigeminal nerve pathways circumventing the BBB (Trevino et al., 2020). The axonal transport though the trigeminal or olfactory nerves is the major pathway for nose-to-brain transport and additionally, diffusional influx into the brain also contributes to this transport (Dhuria et al., 2010; Kamei et al., 2017). The olfactory pathway is occured in the upper region of the nasal route, the olfactory region, and is stretched to the olfactory bulb while trigeminal transport pathway extends to the brain stem (Dhuria et al., 2010). Following olfactory pathway, drugs travel along the axon of the neuron and pass through the nerve bundle that crosses the cribriform plate, and eventually enter the olfactory bulb located on the surface of the brain (Selvaraj et al., 2018). Nasally administered drugs can across the epithelium in the lateral respiratory region and then to the pons through the trigeminal nerve (Thorne et al., 2004; Crowe and Hsu, 2022) through the transportation along the axonal route of trigeminal nerves (Selvaraj et al., 2018).

After the passage through the mucus, paracellular and transcellular transport and extracellular diffusion can mediate the access to the CNS, which can occur in the epithelium of respiratory and olfactory mucosa (Dhuria et al., 2010). Drugs that pass through the lamina propria can diffuse into the perineural space, and then eventually be transported to the CSF (Lochhead and Davis, 2019). After the localization of molecules in the CSF, olfactory bulb, and brain stem, they are transferred to the parenchymal regions of the brain (Dhuria et al., 2010).

## 5 Methodology for CPP-mediated transepithelial delivery

CPPs have emerged as novel vectors in promoting the transepithelial delivery of bioactive molecules, such as peptides and proteins, nucleic acids, and drug carriers like nanoparticles and liposomes (Khafagy et al., 2009a). It is likely that polarized epithelial cells use similar transduction pathways that have been observed in non-polarized cells (Kristensen and Nielsen, 2016). Also, CPP-mediated transepithelial delivery is affected by multiple factors, including the types of CPP and its concentrations, cell types, nature of cargo, experimental conditions, linking methods, and formulations (Kristensen and Nielsen, 2016).

### 5.1 Types of CPPs and cargos in the transepithelial transport

Several CPPs, including TAT-PTD, polyarginine, penetratin, transportan, and their derivatives, have been employed for the drug delivery through the nasal and intestinal epithelium. TAT-PTD, penetratin and polyarginine are generally categorized as cationic CPPs (Kristensen and Nielsen, 2016). More recently, our research group identified a novel hydrophobic CPP derived from the human TCTP (TCTP-PTD) and demonstrated its potential applicability for transepithelial delivery by nasal administration (Kim et al., 2011a; Bae and Lee, 2013; Bae et al., 2018a). TAT-PTD was the first CPP that was used as a peptide vector for *in vitro* insulin delivery across the intestinal epithelium (Liang and Yang, 2005). Insulin chemically linked to TAT-PTD (CGGGYGRKKRRQRRR) showed 6-8 times higher absorption of insulin compared to insulin in the Caco-2 monolayer system, *via* active and transcytosis-like mechanisms (Liang and Yang, 2005). However, some studies reported the inability of TAT-PTD to translocate monolayers of Caco-2 and MDCK epithelial cells (Violini et al., 2002), and in intestinal insulin absorption from the ileum in rats (Kamei et al., 2008). Therefore, polyarginine and penetratin and their analogs have often applied for epithelial drug delivery. An *in vitro* study of epithelial transport of insulin by D-form of arginine octamer (D-R8) indicated that cellular uptake is the critical step for efficient epithelial absorption and showed that D-R8 mediates intestinal epithelial transport of insulin by energy-independent unsaturable uptake (Kamei et al., 2013b).

Biotinylated transportan and its analog transportan 10 were reported to pass through epithelial Caco-2 monolayer cells mainly *via* transcellular transport and partly *via* paracellular pathway, while penetratin passes across cell monolayers in low yields, possibly due to partial degradation (Lindgren et al., 2004). Fluorescein-labeled D-R6 translocates into the rat ileal membranes *via* energy-dependent pathway, involving electrostatic interactions between anionic proteoglycans in membrane and cationic D-R6 peptide. Such interactions initiate endocytic pathways (Kamei et al., 2009), suggesting that intermolecular binding between CPP and cargo is important for CPP-aided intestinal absorption of proteins and peptides. D-R6 enhanced the transepithelial uptake of insulin but not of leuprolide because insulin is negatively charged and associates with cationic R-D6 (Kamei et al., 2009). Importantly, the strength of interaction between CPP and cargo positively correlates with the delivery efficiency across the nasal mucosa of rats (Khafagy et al., 2010b).

### 5.2 Sequence and stereochemical requirements of CPPs

Studies showed that chain length, amphipathicity, hydrophobicity, and basicity of CPPs influence intestinal permeation of insulin (Kamei et al., 2013a). Specifically, not only hydrophobic Trp but also positively charged Arg and Lys residues are important for transmucosal delivery of peptides and proteins (Kamei et al., 2013a). For the complex of penetratin with insulin in transepithelial transduction through the intestinal epithelium in rats, the presence of Arg residue in the peptide was found to be a prerequisite for facilitating transepithelial insulin transport (Kristensen et al., 2015b). Also, the stereochemistry change of penetratin from L- to D-form showed differential effects according to the cargos because IFN-β with D-penetratin showed elevated intestinal and nasal mucosal absorption compared to that of L-penetratin but this phenomenon was not observed in the cases of exendin-4 and glucagon-like peptide 1(GLP-1) delivery in rats (Khafagy et al., 2009b). In most cases, CPP-mediated nasal delivery of insulin and exendin-4 utilized the L-form of penetratin, TCTP-PTD, and their variants because it showed better efficiency than D-forms.

### 5.3 Linking methods for CPPs and cargos

CPPs can be linked to cargos *via* covalent conjugation or noncovalently using a simple mixing method or can be expressed as a fusion protein. For D-R9 and insulin complexation, covalent conjugation of D-R9 to insulin, not their coadministration, is necessary for the translocation through rat pulmonary alveolar epithelium (Patel et al., 2009). D-R9-insulin conjugates showed enhanced hypoglycemic effects in diabetic rats following pulmonary administration. However, coadministration of D-R9 and insulin facilitated the intestinal absorption of insulin (Morishita et al., 2007). In addition, the efficiency of epithelial transport can also be influenced whether CPP is conjugated to the N- or C-terminus in the complex. For the delivery of the active fragment of parathyroid hormone (PTH 1–34), conjugation at N-terminus showed better efficiency than C-terminal conjugation, but coadministration method was more efficient for the delivery of PTH 1-34 in the Caco-2 monolayer (Kristensen et al., 2015a).

Coadministration of CPPs with cargos sometimes forms CPP-cargo complexes *via* intermolecular interactions (Khafagy et al., 2010b). Such interactions include hydrophobic and electrostatic interactions, and hydrogen bonding, which are determined by the factors, including the molecular nature of CPP and cargo. It is generally accepted that not only the binding efficiency between CPP and cargo but other factors collectively contribute to and determine the absorption efficacy (Khafagy et al., 2010b). Other studies warn that covalent linking or fusion of cargo with CPP has the potential to interfere with the natural functions of the cargo (Kristensen et al., 2015a). Most studies on the nasal delivery of antidiabetic peptides, such as insulin, exendin-4, and GLP-1 with CPPs confirmed the effectiveness of the simple physical mixing of CPP with cargos *in vivo*.

### 5.4 Advanced strategies for enhanced transepithelial transport using CPPs

For physical mixing methods, the extent of intermolecular interaction between CPP and cargo is a key determinant for efficient delivery, as shown for penetratin and its analogs (Khafagy et al., 2010b). The strategies for promoting the permeation of the transepithelial layer include conjugation of additional moieties to CPP that enhance the intermolecular interactions, mucus permeation, and adhesion. Conjugation of penetratin with bis-β-cyclodextrin (bis-CD), a cyclic oligosaccharide, improved assembly of insulin into nanocomplexes, and penetratin-bis-CD enhanced epithelial translocation of coadministered insulin compared with that of penetratin (Zhu et al., 2014). Such enhancement was due to the elevated hydrophobicity by bis-CD conjugation, which reinforced electrostatic and hydrophobic interactions between insulin and nanocomplexes (Zhu et al., 2014). Furthermore, penetratin-bis-CD tends to retard the proteolysis of insulin compared to that of penetratin alone (Zhu et al., 2014). Similarly, the conjugation of stearic acid to the R6EW (SA-R6EW), a R7E and R8W substituent of R8, considerably enhanced intestinal delivery of insulin in rats compared to that of R8 *via* enhanced intermolecular interactions in the complex (Zhang et al., 2015).

Poly (N-(2-hydroxypropyl) methacrylamide polymer) (pHPMA)-coated penetratin-insulin complex that has almost neutral surface charge showed enhanced mucus diffusion and insulin delivery in a diabetic rat model compared to nanoparticles whose surface charge is negative (Shan et al., 2015). pHPMA-coating induced the facilitated mucus permeation and adhesion by reducing the potential electrostatic interaction with mucus constituents (Shan et al., 2015). Also, nanoparticles combined with anionic chitosan showed enhanced paracellular penetration in the mucosal delivery by facilitating mucosal adhesion (Ozsoy et al., 2009; Casettari and Illum, 2014). Chitosan has been widely used as an intranasal absorption enhancer for various therapeutics and is a mucoadhesive agent because amines in chitosan mediate its interaction with sialic acid expressed on the mucosal layer (Ozsoy et al., 2009; Casettari and Illum, 2014). Chitosan, a polycationic polymer that has positive charge, enhances paracellular transport by inducing the structural reorganization of tight junction-associated proteins (Schipper et al., 1997). It was found to reduce mucociliary clearance from the nasal cavity and to open tight junctions in the olfactory epithelium (Ozsoy et al., 2009; Casettari and Illum, 2014).

## 6 CPP-mediated systemic delivery of antidiabetic peptides through nasal administration

Therapeutic proteins and peptides can be administered parenterally by intravenous or subcutaneous routes. Such large molecules are required to cross the extracellular spaces for delivery through the circulation (Dinca et al., 2016). If the drugs cannot be administered systemically, nasal administration route may be a possible alternative because it can avoid the first-pass metabolism and initiate rapid action. CPPs were reported to facilitate the nasal delivery of non-injectable biotherapeutics (Kristensen and Nielsen, 2016) for release into the systemic circulation (Dinca et al., 2016).

Conjugation of CPPs to cargos or coadministration of CPPs with the molecules can increase cellular absorption and bioavailability of otherwise nonpenetrating drugs (Maeng and Lee, 2022). Coadministration of CPP with cargo is preferred because of the simplicity of the application method, and the *in situ* release of the cargos from the complex, maintaining its natural conformation. Various peptides, such as insulin, exendin-4, and GLP-1 with CPPs, including TCTP-PTD, penetratin and their variants nasally coadministered were studied to confirm their systemic delivery (Table 2).

**TABLE 2:** Nasally delivered systemic antidiabetic peptides using CPPs.

PTD	Methodology	*In vivo* system	Findings	Reference
Insulin				
TCTP-PTD 13	Simple mixing	STZ-induced diabetic mice	The pharmacological BA (F%) of nasal insulin/L-TCTP-PTD 13 relative to s.c. insulin was 21.3%	Bae and Lee, (2013)
TCTP-PTD 13M2	Simple mixing	Alloxan-induced diabetic rat	The F% of L-TCTP-PTD 13M2/insulin was about 42.3%, which was higher than that of L-TCTP-PTD 13/insulin (22.4%)	Bae et al. (2018b)
Penetratin	Simple mixing	Normal rats	The pharmacological availability and BA of nasal L-penetratin/insulin was 76.7% and 50.7% respectively, relative to the s.c. insulin	Khafagy et al. (2009a)
Shuffle (R,K fix) 2	Simple mixing	Normal rats	Shuffle (R,K fix) 2 significantly enhanced the systemic delivery of coadministered insulin, reaching relative BA value 1.85-times that of penetratin (19.8% vs. 36.7%)	Khafagy et al. (2010a)
PenetraMax	Simple mixing	Normal rats	One-month twice daily nasal administration with PenetraMax resulted in an increase of insulin BA up to 2.2 times compared to that of L-penetratin (48.8% vs. 105.0% at 2.0 mM CPPs)	Khafagy et al. (2013)
Exendin-4				
TCTP-PTD 13M2	Simple mixing	Normal rats	The relative BA of nasal TCTP-PTD 13M2/exendin-4 was 31.5% compared with s.c. exendin-4 in normal rats	Bae et al. (2018a)
	Simple mixing	Type 2 *db/db* mice	TCTP-PTD 13M2 decreased blood glucose level by 43.3% compared with that of exendin-4 in *db/db* mice	Bae et al. (2018a)
TCTP-PTD 13M3	Simple mixing	Normal rats	The relative BA of nasal TCTP-PTD 13M3/exendin-4 was 29.3% compared with s.c. exendin-4 in normal rats	Bae et al. (2018a)
Penetratin	Simple mixing	Normal rats	The BA of intranasally coadministered L-penetratin with exendin-4 was 7.7%	Khafagy et al. (2009b)
Shuffle (R,K fix) 2	Simple mixing	Normal rats	Higher plasma exendin-4 concentration by coadministration of exendin-4 with shuffle (R,K fix) 2 than that of L-penetratin in normal rats	Khafagy et al. (2010b)
GLP-1				
Penetratin	Simple mixing	Normal rats	The BA of intranasally coadministered L-penetratin with GLP-1 was 15.9%	Khafagy et al. (2009b)
Shuffle (R,K fix) 2	Simple mixing	Normal rats	Higher plasma GLP-1 concentration by coadministration of GLP-1 with shuffle (R,K fix) 2 than that of L-penetratin in normal rats	Khafagy et al. (2010b)

### 6.1 Systemic insulin delivery using CPPs through the nasal route

Insulin is a polypeptide hormone that circulates in the blood stream to regulate the glucose homeostasis by binding to its cell surface receptor and facilitates glucose uptake into cells (Rahman et al., 2021). Insulin is administered subcutaneously to the patients with diabetes mellitus who have insulin deficiency. It is addressed that pharmacokinetic profile of intranasal insulin mimics that of the endogenous ‘pulsatile’ insulin (Illum and Davis, 1992; Khafagy et al., 2009a). TCTP-PTD and its variants TCTP-PTD 13, 13M2, as well as penetratin and its analogs shuffle (R,K fix) 2 and PenetraMax were tested for the systemic delivery of intranasal insulin.

#### 6.1.1 TCTP-PTD and its variants

Our group previously reported on CPP derived from human TCTP that we called TCTP-PTD (Bae and Lee, 2013; Bae et al., 2019; Kim et al., 2019; Maeng and Lee, 2022). TCTP-PTD, MIIYRDLISH, was identified from the N-terminal 10-amino acid moiety of the full-length TCTP based on the fortuitous finding that full-length TCTP can cross the cells (Kim et al., 2011a). TCTP-PTD also has ability to transport various types of cargo molecules, including peptide, protein, and siRNA, both linked covalently and non-covalently (Kim et al., 2011a; Maeng and Lee, 2022). TCTP-PTD penetrates the cell membrane mainly *via* the mechanism by caveolae-mediated endocytosis and partly through macropinocytosis (Kim et al., 2011a, 2015). Simple coadministration of TCTP-PTD with bioactive peptides and proteins was demonstrated to be an efficient method to deliver them *in vivo* (Kim et al., 2011a; Bae and Lee, 2013; Bae et al., 2018a; 2018b). We also identified the sequence requirements for TCTP-PTD for its translocating ability and constructed some of its variants through the substitution of amino acid residues that exhibit enhanced translocating ability with negligible cytotoxicity (Kim et al., 2011b).

In an earlier study, we tested the feasibility of TCTP-PTD and its variants, TCTP-PTD 3, 8, and 13, as vehicles for the intranasal insulin delivery (Bae and Lee, 2013). Here, insulin was noncovalently linked with TCTP-PTD by physical mixing and tested for its hypoglycemic effects in normal mice. Nasal administration of insulin mixed with TCTP-PTD 13 (MIIFRALISHKK) significantly reduced blood glucose levels while the administration of insulin mixed with TCTP-PTD, TCTP-PTD 3, and 8 did not exhibit significant hypoglycemic effects (Bae and Lee, 2013). The molar ratio between insulin and TCTP-PTD 13 was found to be a critical factor for nasal insulin absorption, and the best ratio was 1:2 (Bae and Lee, 2013). The intranasal insulin delivery by TCTP-PTD 13 was also confirmed in streptozotocin (STZ)-induced diabetic mice. The pharmacological BA (F%) of nasal insulin/TCTP-PTD 13 relative to subcutaneous administration was 21.3% (Bae and Lee, 2013). When the mucosal penetration of insulin/FITC-labeled TCTP-PTD 13 was visualized in mice with STZ-induced diabetes, TCTP-PTD 13 rapidly penetrated the nasal epithelium after nasal administration, and then was partially distributed in the submucosal layer (Bae and Lee, 2013). The nasal insulin/TCTP-PTD 13 did not cause any nasal mucosal damage in STZ-induced diabetic mice after administration for 7 days (Bae and Lee, 2013). When the nature of the molecular interaction between insulin and TCTP-PTD 13 was investigated using fluorescence resonance energy transfer (FRET), it showed significant FRET signals, indicating that their noncovalent intermolecular interaction are in proximity (Bae and Lee, 2013).

Next, we examined the ability of the variants of TCTP-PTD 13 to enhance the efficiency of intranasal insulin delivery. As overall hydrophobicity of TCTP-PTD variants (Kim et al., 2011b) and 1-MIIFR-5 and 9-SHKK-12 residues of TCTP-PTD 13 are favorable for intranasal transduction and solubility, the residues 6-ALI-8 were considered for amino acid residue substitution (Bae et al., 2018b). 6-IAA-8 substitution was ruled out because the variant TCTP-PTD 8 (MIIYRIAASHKK) did not exhibit enhanced nasal insulin delivery (Bae et al., 2018b). As for TCTP-PTD 13M1 (MIIFRLLISHKK), a variant having 6-LLI-8 moiety with increased hydrophobicity, it did not exhibit enhanced delivery efficiency compared to TCTP-PTD 13 (Bae et al., 2018b). We speculated that the relatively large side chain of Leu, which can cause steric hindrance, might cause structural changes in the peptide and reduce the solubility (Bae et al., 2018b). Therefore, we designed double substitution of A6L and I8A of TCTP-PTD 13 (TCTP-PTD 13M2, MIIFRLLASHKK) to reduce potential steric hindrance while retaining its overall hydrophobicity. TCTP-PTD 13M2 was taken up by cells more efficiently with an enhanced pharmacokinetic (PK) profile in normal rats than that of TCTP-PTD 13. The relative BAs of intranasally administered (i.n.) insulin, insulin/TCTP-PTD 13, and insulin/TCTP-PTD 13M2 were 0.4%, 22.1%, and 37.1%, respectively, relative to subcutaneously administered (s.c.) insulin (Bae et al., 2018b). The difference in the amino acid residue at position 8 between TCTP-PTD 13M1 and 13M2 significantly affected the efficiency and solubility of CPP/cargo complex in the nasal delivery. More importantly, nasal TCTP-PTD 13M2/insulin was more effective in lowering blood glucose levels than nasal TCTP-PTD 13/insulin in alloxan-indued diabetic rats with F% of 42.3% and 22.4%, respectively (Bae et al., 2018b). Toxicity analysis revealed that TCTP-PTD 13M2 did not induce the damage of nasal mucosa in normal rats (Bae et al., 2018b). After intraperitoneal injection of L-TCTP-PTD 13M2 for 10 days in normal mice, analysis of blood and urine samples revealed that liver and kidney functions were normal (Bae et al., 2018b).

Interestingly, the altered stereochemistry of TCTP-PTD variants was not necessarily favored because both D-TCTP-PTD 13 and D-TCTP-PTD 13M2 did not exhibit enhanced uptake compared to their L-counterparts (Bae et al., 2018b). Mixing of D-TCTP-PTD variants with insulin appeared to form larger molecular complexes than the mixtures with the L-form’s (Bae et al., 2018b). This was similar to the observation that L-penetratin was more efficient than D-penetration in intranasal insulin delivery (Khafagy et al., 2009a). It appears that a decrease in the insulin release from the insulin/D-penetratin complex was the possible cause of lower efficiency of D-penetratin (Khafagy et al., 2009a).

In order to further optimize the efficacy of CPP-aided nasal insulin delivery, we conducted formulation studies of TCTP-PTD 13 and 13M2 (designated as PTD 1 and 2, respectively) (Kim et al., 2019). TCTP-PTD 13M2 showed higher compatibility with insulin in terms of biophysical aspects and stable complex formation but exhibited higher aggregation (Kim et al., 2019). Arginine hydrochloride (Arg HCl) and glycerin were used as aggregation inhibitor and osmolyte, respectively, in the formulation. The relative BA of the intranasally administered TCTP-PTD 13M2/insulin mixture supplemented with 100 nM Arg HCl and 16 mg/ml glycerin was 58% and that of TCTP-PTD 13M2/insulin containing 25 mM Arg HCl and 1 w/v% sucrose was 53%, relative to the BA of one that was administered subcutaneously (Kim et al., 2019). The PK profile of intranasal absorption of insulin depends on the concentration of carbohydrate in the formulations (Kim et al., 2019).

The above formulations supplementing with Arg HCl and sucrose or glycerin showed enhanced BA and blood glucose-lowering efficacy *in vivo* (Kim et al., 2019), but Arg HCl was reported to have dual effects on the protein aggregation (Smirnova et al., 2015; Borzova et al., 2017). Our group performed additional formulation studies to further enhance the BA of nasal insulin/TCTP-PTD 13M2 and to delineate the storage conditions of the formulation (Bae et al., 2019). Eight different formulations were constructed by adjusting the pH, and concentrations of sucrose and CPP, but all contained Poloxamer 188 as a surfactant and 1 mM methionine as an antioxidant (Bae et al., 2019). Of the formulations, BAs of formulations 3-3 and 3-5 were about 61% and 46%, respectively, relative to s.c. insulin that was higher than that of insulin/TCTP-PTD 13M2 without formulation (about 39%) in normal rats (Bae et al., 2019). In alloxan-induced diabetic rats, both formulations showed a hypoglycemic effect that lasted for 240 min, while i.n. insulin showed negligible hypoglycemic effects (Bae et al., 2019). The pharmacological availability values of 3-3 and 3-5 formulations that were calculated relative to s.c. insulin were about 49% and 38%, respectively, whereas that of insulin/TCTP-PTD 13M2 was only 4% (Bae et al., 2019). Both 3-3 and 3-5 formulations contain 0.1 mM insulin, 0.25 mM TCTP-PTD 13M2, 1% sucrose, 0.5 mg/ml Poloxamer 188, and 1 mM methionine without Arg HCl, but they only differ in the pH (pH 6.4 and 7, respectively) (Bae et al., 2019). Stability analysis of TCTP-PTD 13M2/insulin mixture in 3-3 formulation found that 96 h storage at room temperature decreased the area under the curve (AUC) value by 77% whereas storage of formulation 3-3 at 4°C maintained its efficiency for up to 7 days with AUC value at about 85% *in vivo* (Bae et al., 2019). Also shown was that insulin is more susceptible to proteolytic degradation than TCTP-PTD 13M2 peptide and that insulin aggregates formation after the prolonged time of storage results in the decreased efficiency of nasal insulin delivery (Bae et al., 2019).

Taken together, physical mixing of TCTP-PTD variants with insulin was demonstrated to be an efficient and safe strategy in the nasal insulin delivery in diabetic murine models. The formulation studies indicated the possibility of more enhanced efficiency of nasal insulin delivery by TCTP-PTD variants with a BA of 61%, indicating the potential application of CPP-aided nasal insulin delivery serving as an alternative route for the invasive s.c. insulin.

#### 6.1.2 Penetratin and its variants

A series of studies by another group indicated the usefulness of penetratin in the nasal delivery of antidiabetic peptides. Penetratin, a cationic CPP (RQIKIWFQNRRMKWKK) with a hydrophobic and amphipathic nature, is derived from *Drosophila* homeodomain and is mainly internalized into cells *via* macropinocytosis (El Andaloussi et al., 2007). The penetratin’s amino acid sequence is characterized by a high content of basic amino acids like Arg and Lys and by the presence of hydrophobic Trp. The intermolecular interaction between penetratin or its analogs and coadministered cargo is essential for the CPP-aided intranasal delivery of the cargo *in vivo* (Khafagy et al., 2010b). The hydrophobic nature of penetratin was proposed as the primary prerequisite for its cell membrane penetration, together with intermolecular hydrogen bonding in its secondary structure (Khafagy et al., 2009b). Further studies on the sequence requirements for intranasal transport and *in silico* analysis identified the moieties essential for penetratin’s carrier ability and the variants, including shuffle (R,K fix) 2 (Khafagy et al., 2010b) and PenetraMax (Khafagy et al., 2013).

Khafagy et al. (2009a) reported that mixtures of CPPs, including L- or D-forms of R8 and penetratin, respectively, with insulin, facilitated nasal absorption in rats, and L-penetratin showed the best efficiency. Both L- and D-forms of penetratin dramatically enhanced nasal insulin absorption when administered as a physical mixture. Coadministration of L-penetratin with insulin dose-dependently elevated pharmacological availability (PA) and BA up to 76.7% and 50.7%, respectively, relative to the s.c. insulin in normal rats whereas insulin alone did not exert a hypoglycemic effect (Khafagy et al., 2009a). However, the increase in D-penetratin concentration reduced nasal absorption of insulin (Khafagy et al., 2009a). L-penetratin did not induce the damage of cellular integrity in nasal mucosa (Khafagy et al., 2009a). In spite of more resistant biochemical properties of D-form against proteases and peptidases, L-penetratin showed better efficiency than D-penetratin in nasal insulin delivery. This can be explained by the observation that even partially fragmented or modified penetratin retained penetrating ability and exhibited its efficacy (Brattwall et al., 2003; Khafagy et al., 2009a). Interestingly, the concentration of L-penetratin appears to determine the delivery activity *via* the interaction between L-penetratin and plasma membrane (Khafagy et al., 2009a). Electrostatic and hydrophobic interactions between hydrophobic (Trp) or basic amino acids of penetratin and negative charges of hydrophobic glycosaminoglycan (GAG) components of the plasma membrane facilitate the transepithelial delivery in the nasal cavity (Christiaens et al., 2004).

Since penetratin has the disadvantages that relatively high doses of penetratin are required for nasal insulin absorption (Khafagy et al., 2009a), several analogs of penetratin were designed looking to optimize the efficacy of transepithelial insulin delivery. Based on the sequence requirements in penetratin for transduction, such as hydrophobicity, amphipathicity, basicity, and the chain length (Khafagy et al., 2010a), a modified peptide designated as shuffle (R,K fix) 2 (RWFKIQMQIRRWKNKK) that exhibits safe and enhanced insulin delivery was developed (Khafagy et al., 2010a). The positions of amino acid residues in penetratin (RQIKIWFQNRRMKWKK) were shuffled, while those of Arg and Lys residues were fixed in the sequence (Khafagy et al., 2010b). Shuffle (R,K fix) 2 showed a significant enhancement in the delivery of coadministered insulin through the rat nasal cavity, reaching a relative BA value of 1.85-folds that of L-penetratin/insulin (19.8% vs. 36.7%) (Khafagy et al., 2010a). This variant was also found to be effective in nasal absorption of other antidiabetic peptides, such as GLP-1 and exendin-4 (Khafagy et al., 2010b). The binding affinities between shuffle (R,K fix) 2 and cargos, including insulin, exendin-4, and GLP-1, were higher than those of L-penetratin (Khafagy et al., 2010b). The higher degree of binding, expressed as the binding ratio between shuffle (R,K fix) 2 and insulin or GLP-1, was found to be higher than that of L-penetratin, indicating that the level of nasal absorption is largely determined by the binding ratio between CPP and drugs (Khafagy et al., 2010b).

Additional *in silico* analysis using molecular orbital study with self-organizing map (SOM) classification, identified the novel penetratin analog named PenetraMax (KWFKIQMQIRRWKNKR) (Khafagy et al., 2013). It showed stronger interaction with insulin as well as more facilitated absorption of insulin than that of penetratin following acute or subchronic administration in normal rats (Khafagy et al., 2013). A single nasal coadministration of PenetraMax with insulin resulted in a 1.9–2.3 times elevation of nasal insulin BA, at different concentrations of CPPs (0.5 vs. 2.0 mM), compared with nasal L-penetratin/insulin (Khafagy et al., 2013). Moreover, consecutive administration for 7 days of intranasal PenetraMax with insulin increased BA of nasal insulin up to 1.8–2.5 times than that of nasal L-penetratin/insulin (Khafagy et al., 2013). After twice daily nasal administrations for 1 month, PenetraMax elevated the insulin BA up to 2.1–2.2 times than that of L-penetratin in rats (for example, 48.8% vs. 105.0% at 2.0 mM CPP) (Khafagy et al., 2013). Of note, nasal PenetraMax/insulin administration showed significantly higher BA that is almost 100% relative to subcutaneous insulin injections after acute and subchronic administration (Khafagy et al., 2013). It was found that nasal mucosal toxicity and release of inflammatory and immunogenic mediators in plasma were negligible following nasal PenetraMax with or without insulin after acute and subchronic administration (Khafagy et al., 2013). Histopathological studies showed that there was no significant change in membrane integrity after administration of PenetraMax and L-penetratin, with or without insulin, for 30 consecutive days (Khafagy et al., 2013).

It was suggested that improved efficiency of Shuffle (R,K fix) 2 and PenetraMax is due to the rearrangement of hydrophobic Trp residues (Lensink et al., 2005; Khafagy et al., 2010a, 2013). Consistently, it was suggested that the presence of Trp residue rather than overall hydrophobicity of peptides affects the internalization of amphipathic CPPs (Bechara et al., 2013). As for PenetraMax, enhanced conformational flexibility of the residues enabled the stabilization of peptide conformation, which can promote the membrane insertion of PenetraMax (Polyansky et al., 2009). It was noted that Trp, Lys, Arg backbone spacing may significantly affect the delivery efficacy of CPP, similar to the report that Trp content and backbone spacing are important for its uptake mechanism and efficiency (Rydberg et al., 2012).

### 6.2 Systemic exendin-4 and GLP-1 delivery using CPPs through the nasal route

Glucagon-like peptide-1 (GLP-1), an endogenous 30-amino acid peptide hormone that is mainly produced by intestinal L-cells in response to ingestion of nutrients, plays a role in the regulation of glucose homeostasis (Runge et al., 2007). Exendin-4, a homolog of GLP-1 acting as an agonist for the GLP-1 receptor, is a 39-amino acid peptide that was initially identified in the venomous lizard *Heloderma suspectum* (Runge et al., 2007). Both GLP-1 and exendin-4 are antidiabetic peptides useful for treating type 2 diabetes that bind to and activate GLP-1 receptor thereby facilitating glucose-induced insulin secretion. Endogenous GLP-1 is susceptible to cleavage by dipeptidyl peptidase IV (DPP-IV) whereas exendin-4 resists DPP-IV-induced peptide degradation (Runge et al., 2007). TCTP-PTD variants, such as TCTP-PTD 13M2 and 13M3, and penetratin and its analog shuffle (R,K fix) 2 were used in nasal administration of exendin-4 and GLP-1 *via* physical mixing method and were verified to be effective in normal or diabetic murine models.

#### 6.2.1 TCTP-PTD variants

We investigated nasal exendin-4 delivery by several TCTP-PTD variants and their hypoglycemic responses in normal rats and in diabetic mice, respectively. The variants include TCTP-PTD 13, 13M1, 13M2, and 13M3 (MIIFRLLAYHKK). TCTP-PTD 13M3 is a novel variant with S9Y substitution of TCTP-PTD 13M2, which was designed to enhance the hydrophobicity of peptide and its solubility by the hydroxyl group of Tyr (Bae et al., 2018a). Nasal coadministration of exendin-4 with TCTP-PTD 13, 13M1, 13M2, and 13M3 showed that TCTP-PTD 13M2 was the best CPP for intranasal exendin-4 delivery in normal rats, and its effectiveness followed the following order: TCTP-PTD 13M2, 13M3, 13M1, and 13 (Bae et al., 2018a). TCTP-PTD 13M2 and 13M3 exhibited almost comparable PK parameters with relative BAs of 31.5% and 29.3%, respectively (Bae et al., 2018a). The reinforced hydrophobicity in TCTP-PTD 13M3 increased exendin-4 nasal delivery but it was not more efficient than TCTP-PTD 13M2 (Bae et al., 2018a). Hypoglycemic effect of nasally administered TCTP-PTD 13M2/exendin-4 was superior to that of nasal TCTP-PTD 13/exendin-4 in type 2 *db/db* mice (Bae et al., 2018a). It was shown that nasal TCTP-PTD 13M2/exendin-4 decreased blood glucose levels by 43.3%, compared with that of exendin-4 alone and by about 18.6% compared with that of TCTP-PTD 13/exendin-4 in *db/db* mice (Bae et al., 2018a). Neither TCTP-PTD 13 nor 13M2 caused nasal mucosal damage (Bae et al., 2018a).

Additionally, we examined whether the linking methods affect the intranasal delivery of exendin-4 *in vivo*. TCTP-PTD 13M2 peptide was covalently fused to the N- or C-terminus of exendin-4 (N-M2-Exendin-4 and C-M2-Exendin-4, respectively) *via* three Gly linker, which were placed between them to minimize the potential steric hindrance between CPP and cargo (Bae et al., 2018a). Subcutaneous injection of both N-M2-Exendin-4 and C-M2-Exendin-4 in *db/db* mice showed a lesser effect than s.c. exendin-4 though C-M2-Exendin-4 was superior to that of N-M2-Exendin-4 (Bae et al., 2018a). When the hypoglycemic effect of C-M2-Exendin-4 was compared with that of a physical mixture of TCTP-PTD 13M2/exendin-4 after nasal administration in *db/db* mice, C-M2-Exendin-4 exhibited much lower efficiency than that of TCTP-PTD 13M2/insulin (Bae et al., 2018a). The instability of the fusion peptide under the physiological conditions in the nasal cavity was suggested as one potential mechanism for the reduced efficacy of the conjugated complex (Bae et al., 2018a). Therefore, the physical mixing method can be preferentially applicable to the intranasal delivery of antidiabetic peptides, such as exendin-4 *in vivo* (Bae et al., 2018a).

#### 6.2.2 Penetratin and its variants

Transepithelial delivery of exendin-4 through the nasal mucosa by penetratin was found to be effective *in vivo* (Khafagy et al., 2009b). When the efficiencies of L-penetratin-aided exendin-4 deliveries by nasal and intestinal mucosal route were compared, the nasal route was more effective in the delivery of GLP-1 and exendin-4, than that of absorption from ileal loop (Khafagy et al., 2009b). The BAs of intranasally coadministered L-penetratin with GLP-1 and exendin-4 were 15.9% and 7.7%, respectively, whereas BA of intestinally coadministered L-penetratin with GLP-1 and exendin-4 was 5% and 1.8%, respectively, in rat ileal loops (Khafagy et al., 2009b). The variance between nasal and intestinal BAs might be due to the differences in the first-pass metabolism following the administration of peptides *via* the intestinal loop and by the unequal enzymatic activities in the nasal and intestinal epithelium (Khafagy et al., 2009b). D-penetratin however did not exhibit any significant effect on the nasal delivery of GLP-1 and exendin-4 *in vivo*, indicating the effect of stereochemistry on the transepithelial penetration of CPP (Khafagy et al., 2009b).

GLP-1 has a low isoelectric point (pI), and therefore, the negative charge of GLP-1 at the administration site was postulated to contribute to its association with positively charged penetratin. Biochemical properties of penetratin also facilitate its binding to membrane components, including anionic phospholipids and GAGs, through electrostatic interaction (Poon and Gariépy, 2007; Khafagy et al., 2009b). Exendin-4 shares sequence similarity with GLP-1 exhibiting a low pI (pI 4.5) in physiological fluids (Khafagy et al., 2009b). It is likely that exendin-4 exerts electrostatic or hydrophobic interactions *via* its negatively charged residues with basic and hydrophobic residues of penetratin (Khafagy et al., 2009b).

As mentioned above, the penetratin analog, shuffle (R,K fix) 2, exhibited a more significant hypoglycemic effect compared with L-penetratin after intranasal administration of insulin, exendin-4, and GLP-1 in normal rats (Khafagy et al., 2010b; 2010a). The binding affinities between GLP-1 or exendin-4 and shuffle (R,K fix) 2 were much higher than those of L-penetratin (Khafagy et al., 2010b). Calculated binding ratios between bound concentrations of CPP and immobilized peptide drugs at nasal absorption conditions revealed that binding ratios between L-penetratin and GLP-1 and exendin-4, respectively, were elevated when shuffle (R,K fix) 2 was used, indicating that the intermolecular interaction between CPP and cargo is critical for nasal peptide drug absorption (Khafagy et al., 2010b).

## 7 CPP-mediated nose-to-brain delivery of antidiabetic peptides

Alzheimer’ disease (AD) is a progressive and degenerative cerebral disorder accompanied by cognitive decline. Recently, growing evidence supports the concept that AD is a metabolic disease of type 3 diabetes mellitus, based on the fact that insulin resistance and dysfunctional glucose metabolism in the brain are often found in patients with AD and that Aβ deposition induces reduced expression of insulin receptor on neuronal cells (Zhao et al., 2008; de la Monte and Tong, 2014; Butterfield and Halliwell, 2019; Kellar and Craft, 2020). Insulin signaling in the brain regulates memory and cognitive functions as a neuromodulator in hippocampal neurons *via* the insulin receptor pathway, whereas insulin resistance in the brain is a pathophysiological hallmark of AD (Stanley et al., 2016; Hallschmid, 2021). Insulin administration reduces insulin resistance (Akhtar and Sah, 2020) and directly regulates CNS function through the insulin receptor (Havrankova et al., 1978). In this context, insulin signaling is regarded as a novel target in the therapy of memory loss in AD (Hallschmid, 2021).

More recently, it has been demonstrated that CPPs can cross the biological barriers including BBB, retina and neuron, intestinal wall, and skin that are otherwise difficult to penetrate by conventional delivery systems, especially for the delivery of macromolecules (Lin et al., 2016). Because drugs must penetrate the nasal epithelium before their uptake into neuronal and supporting cells, CPPs can be used for enhancing nasal delivery. With the aid of CPP-mediated transport, nose-to-brain delivery can be achieved by minimizing the significant systemic exposure of administered drugs. CPPs can facilitate the direct nose-to-brain delivery of drugs, which might help reduce the administration doses to reach the therapeutic concentration of drugs in the brain. For the therapy of AD, antidiabetic peptides, such as insulin and GLP-1 receptor agonist, have been tested for their potential in promoting memory and learning functions by facilitating the insulin receptor signaling and glucose transport in hippocampal neurons (Kamei et al., 2018). Among the great variety of CPPs, penetratin was reported to be effective in the nose-to-brain delivery of antidiabetic peptides, including insulin and exendin-4, in an animal model of AD (Table 3).

**TABLE 3:** Nose-to-brain delivery of antidiabetic peptides using CPPs.

PTD	Methodology	*In vivo* system	Findings	Reference
Insulin				
Penetratin	Simple mixing	Normal mice	Coadministration of L-penetratin with insulin facilitated nose-to-brain insulin delivery to the distal regions of the brain.	Kamei and Takeda-Morishita, (2015)
	Simple mixing	SAMP8 mouse model	Chronic nasal insulin/L-penetratin treatment retarded the memory loss in SAMP8 mice at an early stage of cognitive dysfunction but not at the progressive stage.	Kamei et al. (2017)
Exendin-4				
Penetratin	Simple mixing	Normal mice	L-penetratin facilitated the nose-to-brain delivery of exendin-4 in normal mice.	Kamei et al. (2018)
	Simple mixing	SAMP8 mouse model	Nose-to-brain delivery of exendin-4 and insulin with L-penetratin boosted the therapeutic effect of exendin-4 in the progressive memory loss of SAMP8.	Kamei et al. (2018)

### 7.1 Nose-to-brain delivery of insulin using penetratin

Insulin delivery through nose-to-brain pathway is aimed to improve memory by enhancing synaptic plasticity and glucose uptake, thereby attenuating the neuropathy associated with AD (Hallschmid, 2021). Mounting evidence has shown that acute and chronic intranasal insulin administration facilitates memory and recognition functions (Brünner et al., 2013) both in healthy individuals and patients with mild cognitive impairment of AD (Hallschmid, 2021). Because insulin cannot be passively transported into the brain due to its high molecular weight of 5,800 Da (Hallschmid, 2021) and the efficiency of delivery to the brain by intranasal administration is low, CPPs are considered as the ideal potential vehicles (Kamei and Takeda-Morishita, 2015).

Takeda-Morishita group employed the noncovalent method for nose-to-brain delivery of penetratin that was found to be better than octaarginine (R8) in intestinal and nasal delivery (Kamei et al., 2008; Khafagy et al., 2009a). When they compared the absorption and distribution profiles of systemic (i.v.) and intranasal (i.n.) administration of insulin in mice, they found that i.v. insulin was mainly found in the systemic circulation with slight distribution in the brain (Kamei and Takeda-Morishita, 2015). In contrast, the intranasal route enhanced the distribution of insulin to the olfactory bulb with minimal systemic exposure of insulin. As i.n. insulin requires administration of more than 10 times dose than i.v. route for the comparable levels of insulin in the brain, this group employed L- and D-penetratin to maximize the nose-to-brain insulin delivery (Kamei and Takeda-Morishita, 2015). L-penetratin exhibited a more efficient transport of insulin to the mouse brain following i.n. administration than D-penetratin, whereas D-penetratin showed less systemic insulin exposure (Kamei and Takeda-Morishita, 2015).

Higher insulin in the blood did not necessarily correlate with elevated insulin levels in the brain, implying that blood-to-brain delivery is not the main contributor to nasal L-penetratin/insulin delivery (Kamei and Takeda-Morishita, 2015). Coadministration of nasal insulin with L- or D-penetratin mediated the delivery of insulin to the distal regions of the mouse brain, such as the cerebral cortex, brain stem, and cerebellum, possibly from the nasal cavity to CSF and then to the brain regions (Kamei and Takeda-Morishita, 2015). The pathway of peptides from the nasal cavity to the brain regions involves their epithelial uptake through the lamina propria, and influx to CSF passing through the cribriform plate or through the olfactory/trigeminal pathway in the axonal transport pathway (Dhuria et al., 2010). Nasal coadministration of insulin with D- or L-penetratin elevated the hippocampal insulin level both 15 and 60 min after administration, for subsequent delivery into deeper brain regions (Kamei and Takeda-Morishita, 2015). The distribution of insulin in the hippocampal region might be beneficial for improving memory and recognition in the hippocampus (Akhtar and Sah, 2020).

These investigators also assessed the distribution of nasally administered physical mixtures of insulin and L- or D-penetratin and confirmed the direct transport of insulin from the nasal cavity to the brain parenchyma in rats (Kamei et al., 2016). Analysis using a gamma counter and autoradiography of the brain showed the greatest radioactivity in the olfactory bulb and the brain regions close to the administration site at 15 min after nasal administration of radiolabeled insulin with L- or D- penetratin (Kamei et al., 2016). Noncovalent insulin/penetratin complexes effectively deliver insulin to the olfactory bulb or CSF and distal regions of the brain, including the hippocampus through the pathways, the cerebral cortex, cerebellum, and brainstem, possibly by bulk flow or passive diffusion (Kamei et al., 2017). Quantitative analysis of brain CSF samples at 15 min after nasal administration of insulin/L-penetratin showed that insulin levels were increased only in the anterior portion of the CSF, indicating that insulin was distributed to the CSF from the anterior part of the brain near the olfactory bulb (Kamei et al., 2017). These results collectively indicate that penetratin enables direct insulin transport from the nasal cavity to the parenchymal region of the brain (Kamei et al., 2017).

More recently, Kamei *et al.* clarified the transport route of L-penetratin-aided nose-to-brain delivery of insulin and exendin-4 (Kamei et al., 2021). Here, L-penetratin did not facilitate the transport of fluorescence-labeled exendin-4 and insulin *via* the trigeminal pathway. Rather, it shifted their distribution to the olfactory mucosal pathway. The fractions of the drugs that were systemically absorbed or transported through trigeminal pathway did not contribute to the distribution of peptides to the brain. Insulin with L-penetratin diffused to the surface of the olfactory bulb as well as the lower region of the brain around the hypothalamus, possibly from the CSF. Coadministered L-penetratin enabled the peptides to penetrate through the nasal epithelial membrane. Then, the peptides crossed the lamina propria and cribriform plate, thereby reaching the CSF. The peptides were transported from the surface of the olfactory bulb or bottom region of the brain around the hypothalamus to the deeper part of the brain through the CSF or perivascular spaces of the cerebral artery (Kamei et al., 2021).

Intranasal coadministration of noncovalently bound L-penetratin with insulin increased insulin levels in the brain and its pharmacological effects were tested at two differential stages of memory impairment using a senescence-accelerated mouse-prone 8 (SAMP8) model that exhibits spontaneous aging and senescence (Kamei et al., 2017). SAMP8 mice showed age-related cognitive dysfunction with reduced learning ability score as well as elevated amyloid beta (Aβ) concentration in the circulation (Kamei et al., 2017). Chronic nasal insulin/L-penetratin treatment retarded the progression of mild cognitive dysfunction and memory loss in SAMP8 mice at 16–24 weeks of age, an early stage of memory loss (Kamei et al., 2017). However, the severe recognitive dysfunction seen at the progressive stage associated with the deposition of Aβ was not ameliorated following nasal L-penetratin/insulin. Instead, it unexpectedly elevated Aβ plaque accumulation in the hippocampal region of the mice. Likewise, nose-to-brain delivery of L-penetratin/insulin complex was differentially affected according to severity of AD (Kamei et al., 2017). At the early stages of dementia devoid of Aβ accumulation in the brain, nose-to-brain delivery of L-penetratin/insulin appeared to have the therapeutic effect by preventing the mild cognitive dysfunction of early dementia, whereas it did not prevent severe recognitive dysfunction (Kamei et al., 2017).

The investigators suggested that the phenomenon might occur due to the competition between insulin and Aβ for the degradative pathway by insulin-degrading enzyme (IDE), an enzyme involved in the clearance of extracellular Aβ from the brain (Shiiki et al., 2004). Because of such competition for IDE, IDE-mediated Aβ degradation can be inhibited thereby leading to Aβ accumulation in the brain (Kamei et al., 2018). Accumulation of Aβ whether as soluble or insoluble matter inhibits insulin receptor expression on the neurons by increasing the internalization and degradation of the receptors. Therefore, excessive insulin delivery to the brain is not recommended as it may lead to severe recognition impairment due to Aβ plaque formation, corresponding to 33–38 weeks of SMAP8 mice.

### 7.2 Nose-to-brain delivery of exendin-4 and insulin using penetratin

Having established that intranasal insulin/L-penetratin administration was effective at mild stage of cognitive dysfunction but not at severe cognitive dysfunction in senescence-accelerated mice (Kamei et al., 2018), this group investigated the effectiveness of GLP-1 receptor agonist, exendin-4, in modulating severe cognitive impairment. It was previously established that GLP-1 receptor agonists play a role in memory and learning in the brain by stimulating receptor binding and promoting downstream insulin signaling (Kamei et al., 2018). Exendin-4 exhibited a lower risk of hypoglycemia than insulin following excessive systemic exposure.

Intranasal coadministration of exendin-4 with L-penetratin but not with D-penetratin increased exendin-4 distribution throughout the various brain regions, including the olfactory bulb, the entry point for nose-to-brain delivery (Lochhead and Thorne, 2012) and hippocampus, where exendin-4 mediates memory and learning functions after administration in normal ddY mice (Kamei et al., 2018). These observations imply that direct nose-to-brain transport is responsible for the elevated exendin-4 levels in the brain following intranasal coadministration of L-penetratin/exendin-4. However, coadministration of GLP-1 and L-penetratin showed negligible distribution in the olfactory bulb and the whole brain due to elevated systemic absorption (Kamei et al., 2018). Because D-penetratin did not enhance the nose-to-brain delivery of exendin-4 and GLP-1, they suggested that D-penetratin/cargo complex that resists proteolysis, possibly remains in the cytoplasm compared to L-penetratin/cargo complex, and is released less to lamina propria located in the middle part of the olfactory bulb (Kamei et al., 2018). It was also found that nose-to-brain delivery of L-penetratin/exendin-4 can promote the insulin signaling pathway only in the presence of adequate levels of insulin to stimulate insulin receptors in the brain (Kamei et al., 2018). Brain delivery of exendin-4 and insulin by L-penetratin stimulated the downstream signaling of insulin, as shown by Akt phosphorylation in the hippocampus in normal ddY mice (Lalatsa et al., 2014; Kamei et al., 2018).

To test the therapeutic efficacy of nose-to-brain delivery of insulin with exendin-4, exendin-4 and L-penetratin were nasally administered for 30 days with or without insulin at a dose that is adequate to avoid Aβ accumulation in SAMP8 mice (Kamei et al., 2018). Intranasal exendin-4 administration without CPP contributed to learning in the SAMP8 mouse model and coadministration of L-penetratin and insulin improved cognitive function by facilitating the brain delivery of exendin-4 and insulin signaling activation in the brain (Kamei et al., 2018). In addition, nasal administration of exendin-4 contributed to the decrease in Aβ accumulation (Kamei et al., 2018). They speculated that the amount of insulin that does not induce Aβ deposition may induce the limited expression of insulin receptors on the hippocampal neurons under pathological conditions (Kamei et al., 2018). In this situation, exendin-4-mediated GLP-1 receptor stimulation appears to further enhance the initiation of insulin signaling by activating its downstream player, IRS-1 (Kamei et al., 2018). Collectively, these findings suggest that nose-to-brain delivery of L-penetratin/insulin was effective in the early stage of cognitive dysfunction and that nasal administration of L-penetratin/exendin-4 with insulin boosted therapeutic effect of exendin-4 in the progressive memory dysfunction *in vivo*.

## 8 Summary and future perspectives

**FIGURE 3:**
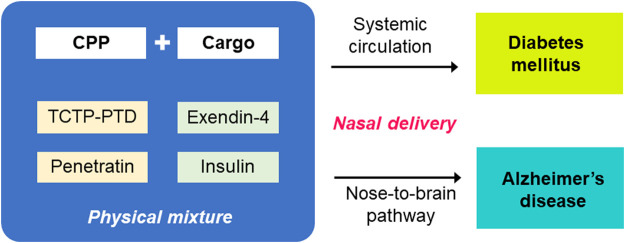
CPP-aided systemic and nose-to-brain delivery of antidiabetic peptides after nasal administration. TCTP-PTD and penetratin and variants thereof shown to be effective in the delivery of antidiabetic peptides, such as exendin-4 and insulin. The *in vivo* efficacy of systemic and nose-to-brain delivery of nasally coadministered CPPs and antidiabetic peptides were confirmed in murine models of diabetic and Alzheimer’s disease, respectively.

Nasal administration of CPP/cargo conjugates or coadministration of CPP with cargo can be employed for systemic and brain delivery of bioactive molecules, if any. Among the various applications for CPP-aided transepithelial transport, antidiabetic peptides, such as insulin, and exendin-4, were studied for its systemic and brain delivery *via* the nasal administration route (Figure 3). Complexes of insulin or exendin-4 with TCTP-PTD and penetratin and their analogs formed using physical mixing method were demonstrated to have the potential applications in the treatment of diabetes or AD. Some variants of TCTP-PTD and penetratin were better than their parent peptides as vehicles for intranasal delivery of antidiabetic peptides.

Discovery of CPPs has opened a way for delivering both small and large molecules into cells and tissues by various methodologies and applications. They were shown to non-toxically penetrate cells, ignoring membrane receptors, thereby avoiding limits on efficiency due to receptor saturation (Ross et al., 2018). Delivery using CPPs is now considered a rational approach in the drug delivery system. TAT-PTD, P28, and activatable CPPs (ACPPs) can be effective armaments in fighting tumors, ocular and inner ear diseases, and Chron’s disease (Xie et al., 2020) among others. The potential disadvantages and challenges in the use of CPPs are *in vivo* instability, cellular toxicity, immunogenicity, non-specific targeting, and endosomal escapes (Zhou et al., 2022).

To deal with some of these challenges, a versatile CPP-based delivery system has been developed and assessed for its effectiveness and safety (Xie et al., 2020). These CPPs can be fusogenic lipids for endosomal escape, novel CPPs for sophisticated and targeted delivery to specific organs and organelles, and CPPs linked to specific ligands such as transferrin, folic acid, specific antibodies, and RGD peptides that increase target specificity (Xie et al., 2020). More specifically, the conjugation of CPPs with polymers or nanoparticles can be considered for improving their stability against proteases, and tissue selectivity (Reissmann and Filatova, 2021). Also, modification of the surface of nanocarriers with CPPs (Kanazawa, 2015) and cell-penetrating homing peptide (CPHP) (Szabó et al., 2022) can assist target cell-specific drug delivery. The smart design of multifunctional nanocarriers has been an area of extensive research because they can be conjugated to endosomal releasing motifs, homing sequences, and activatable sequences at the target sites (Reissmann and Filatova, 2021). Moreover, activatable CPPs enable the specific action of CPPs only when needed because the penetrating ability is hindered by a cleavable linker that is sensitive to specific stimuli, including pH, enzymes, temperature, electricity, and light (Xie et al., 2020). To improve the stability of CPPs, replacement of L-amino acids with D-variants or unnatural amino acids and PEGylation can be considered (Wang et al., 2022). Stapled peptides, cyclization, N-alkylation, and modification of the backbone or side chain are the methods to enhance the stability and internalization of CPPs (Szabó et al., 2022). In terms of endosomal escape, exploitation of “proton sponge effect,” N-terminal stearylation, conjugation of endosomolytic compound, peptides or endosomal escape domains (EEDs) have been studied (Szabó et al., 2022).

It is evident that strategies that optimize the CPP-aided delivery of biotherapeutics are essential for clinical application in conjunction with other drug delivery systems. In this perspective, the precise mechanism of transepithelial uptake and its trafficking of various CPP/cargo complexes or their carrier systems are in great need for the accurate and efficient drug delivery in future studies. During the nose-to-brain delivery, some molecules can be delivered to undesirable regions and cause serious problems because nasally administered drugs can be distributed to both systemic and brain compartments. Therefore, the studies to decipher the contributing factors on the distribution of nasally administered molecules in blood and the brain are necessary for the desirable and safe applications. The multicompartment pharmacokinetic studies with nose-to systemic and nose-to-brain compartments can clearly provide the separation and quantitation of direct nose-to-brain and systemic transport after intranasal administration of the CPP/cargo complex. For the delicate approach in the CPP-aided nasal drug delivery, translational studies on methodologies for the specific targeting of molecules in either systemic or brain regions are essential. The exhaustive efforts to overcome the hurdles in the transepithelial delivery of peptides and proteins can fuel the application of CPP-aided nasal drug delivery in the clinical settings.
